# Perspectives of Kurdish Postnatal Women on Respectful Maternity Care: A Qualitative Study

**DOI:** 10.1002/hsr2.72181

**Published:** 2026-03-22

**Authors:** Khalat Karwan Fares, Hamdia Mirkhan Ahmed, Kochr Ali Mahmood, Sirwan Khalid Ahmed, Hiwa Abdulrahman Ahmad, Moustaq Karim Khan Rony

**Affiliations:** ^1^ Midwifery Department, Koya Technical Institute Erbil Polytechnic University Erbil Iraq; ^2^ College of Health Sciences Hawler Medical University Erbil Kurdistan Region Iraq; ^3^ Faculty of Medicine Koya University Iraq; ^4^ College of Nursing University of Raparin Rania, Sulaymaniyah Kurdistan Region Iraq; ^5^ Department of Medical Microbiology, Faculty of Science and Health Koya University Iraq; ^6^ Miyan Research Institute International University of Business Agriculture and Technology Dhaka Bangladesh

**Keywords:** dignity in childbirth, Kurdish women, maternal health, qualitative research, respectful maternity care

## Abstract

**Background and Aims:**

Respectful maternity care (RMC) is an essential component of high‐quality maternal healthcare and a fundamental human right, ensuring that women experience childbirth with dignity, privacy, and autonomy. Despite global efforts to promote RMC, many women, particularly in low‐ and middle‐income countries, continue to experience mistreatment and disrespect during childbirth. This study aims to elucidate Kurdish postnatal women's preferences regarding respectful behaviors and interactions during labor and delivery, while also exploring their lived experiences, expectations, and the challenges they encounter within the healthcare system.

**Methods:**

A qualitative descriptive study design was employed, from September 18, 2023 to December 26, 2023, utilizing semi‐structured in‐depth interviews with 20 postnatal women in a public hospital setting in the Kurdistan Region of Iraq. Participants were selected through purposive sampling to capture diverse socio‐economic and cultural backgrounds. Thematic analysis was used to identify key themes related to RMC, guided by the WHO framework on respectful maternity care.

**Results:**

Thematic analysis revealed six major themes: respect and dignity, gentleness and supportive care, clear communication, privacy, family presence, and cultural and religious sensitivity. Women emphasized the importance of non‐discriminatory treatment, kind and patient midwives, clear and respectful communication, and privacy during medical procedures. Family presence during labor and culturally sensitive practices were also highly valued. Additionally, some women reported concerns regarding verbal abuse, neglect, and socioeconomic discrimination, indicating gaps in the provision of RMC.

**Conclusions:**

The findings highlight the urgent need for healthcare systems in the Kurdistan region to align maternity care practices with the cultural, emotional, and physical needs of women. Recommendations include enhanced training for maternity care providers on respectful, patient‐centered care, ensuring privacy, accommodating cultural and religious preferences, and supporting the involvement of family members during labor. Addressing these areas can strengthen trust in maternity services, improve maternal experiences, and contribute to broader global goals in maternal health and human rights.

## 1 Introduction

Respectful maternity care (RMC) is widely recognized as a cornerstone of quality healthcare and a fundamental human right during childbirth. It encompasses all aspects of care that uphold women's dignity, privacy, and autonomy, while protecting them from mistreatment, discrimination, and abuse throughout labor and delivery [1]. The World Health Organization (WHO) underscores that RMC is essential to achieving safe, satisfying, and equitable maternal health outcomes. However, individual‐level factors—such as a woman's personal expectations, knowledge of her rights, and prior experiences with the healthcare system—can significantly shape her perception and pursuit of respectful care.

At the interpersonal level, the quality of interactions between patients and providers—including clear communication, informed consent, and shared decision‐making—is crucial in shaping women's maternity experiences. Despite international advocacy initiatives, substantial evidence suggests that disrespect and abuse during childbirth remain prevalent, particularly in low‐ and middle‐income countries [2]. Such experiences may deter women from seeking skilled maternal care in subsequent pregnancies, thereby heightening risks for both mothers and newborns [3].

Community‐level influences further mediate women's maternity care experiences. In the Kurdistan Region of Iraq, sociocultural expectations around gender roles, family authority, and communication norms deeply shape women's autonomy and interactions within healthcare settings  [4]. For instance, cultural beliefs about modesty, gender of the attending provider, or expectations of family involvement may affect how RMC is perceived and experienced. These norms can either support or hinder respectful care depending on how well healthcare practices align with community expectations  [5].

At the structural level, systemic barriers continue to undermine the provision of RMC. These include insufficient resource allocation, overcrowded and understaffed facilities, limited provider training, and inconsistent policy implementation  [6]. While many countries have adopted national and global RMC guidelines, structural deficits in health systems often prevent their effective translation into practice, particularly in fragile healthcare contexts such as that of the Kurdistan Region.

Despite growing global advocacy, there remains a significant gap in understanding how these multi‐level factors shape women's experiences with maternity care in specific cultural and geopolitical settings like Kurdistan region of Iraq. Research in similar contexts has shown that mistreatment manifests in diverse forms, but the Kurdish experience remains underexplored  [7]. Given the region's unique cultural and linguistic context, it is essential to investigate how Kurdish women define and experience RMC.

This research is particularly timely considering international commitments to improve maternal and newborn health, including the United Nations' Sustainable Development Goal (SDG) 3, which emphasizes quality care and well‐being for all  [8]. RMC not only upholds human rights but also fosters trust in healthcare systems, enhances women's satisfaction with childbirth experiences, and promotes positive health‐seeking behaviors [9].

Although global research has increasingly examined the prevalence and impact of disrespect and abuse during childbirth, studies specifically addressing RMC in the Kurdistan Region of Iraq are limited. Existing literature from Iraq has largely focused on clinical quality indicators and maternal outcomes, with little emphasis on women's subjective experiences of care, autonomy, or dignity during labor and delivery [10, 11]. In neighboring regions, studies from Middle East countries have explored elements of respectful care, including communication, cultural sensitivity, and informed consent, yet these findings may not fully translate to the distinct sociopolitical and ethnic context of Kurdistan [12, 13]. To date, no qualitative study has comprehensively examined Kurdish women's perceptions of RMC or explored how cultural, linguistic, and structural factors shape their experiences in public maternity settings. This gap highlights the need for localized research that captures the lived realities of Kurdish women and informs the development of culturally responsive maternity care policies and training.

Therefore, this study aims to elucidate Kurdish postnatal women's preferences regarding respectful behaviors and interactions during labor and delivery, while also exploring their lived experiences, expectations, and the challenges they encounter within the healthcare system. By analyzing these perspectives, the study seeks to identify key barriers and facilitators to the implementation of RMC in the Kurdistan Region. Findings from this research are intended to inform culturally sensitive and patient‐centered improvements in maternity care practices, ultimately contributing to enhanced maternal satisfaction and better health outcomes in the region.

## 2 Methods

### 2.1 Study Design and Setting

This study employed a qualitative descriptive design to explore the perspectives of Kurdish postnatal women regarding RMC. A qualitative approach is particularly valuable in capturing detailed, subjective experiences and providing an in‐depth understanding of participants' perceptions and interactions within maternity care settings [14, 15, 16]. The study started on September 18, 2023 and ended December 26, 2023. The research was conducted in the maternity wards and postnatal care units at Shahid Doctor Khalid Teaching Hospital in Koya, Kurdistan Region of Iraq. The hospital, with a 200‐bed capacity, includes 18 beds specifically designated for labor and delivery and records an average of 150 births per month, covering both vaginal and cesarean deliveries. One of the postnatal ward's forms parts of an integrated unit specifically designed for low‐risk women and their newborns, making it a suitable setting for an in‐depth investigation of maternity care experiences. The choice of this setting ensured accessibility to a diverse range of participants and allowed for a comprehensive exploration of their experiences within a structured healthcare environment.

### 2.2 Study Population and Sampling

The study population consisted of postnatal women who had recently given birth, representing a diverse cohort to capture a broad spectrum of experiences related to maternity care. Participants were selected through purposive sampling, a non‐random technique that ensures the inclusion of women from various age groups, parity levels, socio‐economic backgrounds, and healthcare access points [17]. This strategic selection allowed for a nuanced understanding of individual and collective maternity care experiences. Women were approached and recruited within 1–3 h post‐delivery, before their discharge from the hospital, to ensure that their reflections on labor and delivery were fresh, unfiltered, and accurately recalled. This timing minimized recall bias and provided real‐time insights into their perceptions of maternity care services.

### 2.3 Eligibility Criteria

To ensure the inclusion of participants who could provide meaningful insights into their maternity care experiences, specific eligibility criteria were established. These criteria were designed to maintain the study's focus on women who had undergone postnatal care and could effectively communicate their experiences.

Inclusion criteria: Participants were required to meet the following conditions to be included in the study:

1. Kurdish‐speaking women: As the study was conducted in the Kurdistan Region of Iraq, fluency in Kurdish was essential to ensure clear and accurate communication between participants and researchers.
2. Willingness to participate and provide informed consent: Only women who voluntarily agreed to participate and provided informed consent were included, ensuring ethical adherence and respect for autonomy.

Exclusion criteria: To prevent distress and ensure the appropriateness of participants for the study, the following exclusion criteria were applied:

1. Women experiencing severe postnatal complications requiring immediate or ongoing critical care (e.g., postpartum hemorrhage): Women with serious health conditions necessitating urgent medical attention were excluded to avoid interference with their recovery process.
2. Women who had experienced neonatal loss within the past 24 h: Given the emotional and psychological impact of neonatal loss, these individuals were excluded to avoid undue distress and ethical concerns related to participation.
3. Women unable to understand or communicate in Kurdish: Participants needed to effectively articulate their experiences, and language barriers could hinder the depth and accuracy of data collection.

### 2.4 Data Collection Procedure

Data collection was conducted through in‐depth, semi‐structured interviews in a private setting, ensuring a confidential and comfortable environment for participants. This approach allowed women to openly share their personal experiences, thoughts, and emotions without external pressures or biases. The interviews followed an interview framework based on the WHO model of RMC, which encompasses key domains such as dignity, privacy, communication, and emotional support. By focusing on these aspects, the study sought to uncover the extent to which women experienced respectful and supportive maternity care during labor and delivery. Purposive sampling was employed to ensure diverse representation among participants [17], considering characteristics such as age, parity, socioeconomic status, and accessibility to maternity services. These criteria were identified as relevant to the study objectives and were collected during the initial screening phase through brief pre‐interview demographic forms and verbal screening questions. Participants were recruited with the assistance of maternity care providers and facility staff, who informed eligible women about the study during postnatal follow‐up visits or ward rounds.

To maintain ethical integrity and ensure voluntary participation, all potential participants were approached by a member of the research team only after they were informed by care staff. It was emphasized that participation was entirely optional, would not influence their medical care, and that they could withdraw at any time without consequence. Staff involved in recruitment were trained to avoid coercive language or actions, and no incentives were provided that could unduly influence participation. This process was designed to minimize selection bias and reduce the risk of perceived pressure or obligation to participate.

Interview guide questions

- How did you experience labor and delivery?
- What communication styles by healthcare providers were most helpful to you?
- What aspects of care made you feel respected during labor and delivery?

By using open‐ended questions, participants were encouraged to reflect on their experiences in their own words, fostering rich, qualitative insights that are crucial for understanding the nuances of maternity care. The semi‐structured nature of the interviews also provided flexibility, allowing researchers to probe deeper into specific issues based on participants' responses.

Interviews were conducted in Kurdish by trained researchers proficient in the language and culturally competent, ensuring that participants felt at ease expressing their thoughts and emotions in their native language. Each interview lasted approximately 15–20 min, with audio recording conducted only upon obtaining participant consent. To ensure the integrity and accuracy of data collection, two audio recorders were used simultaneously as a precautionary measure against technical failures. Additionally, field notes were taken to capture non‐verbal cues, emotional expressions, and contextual details, further enriching the qualitative dataset.

### 2.5 Data Analysis

Data analysis followed Ahmed et al. (2025) six‐phase framework for thematic analysis, ensuring a systematic and rigorous approach to identifying key patterns in the data [18]. All interviews were audio‐recorded, transcribed verbatim, and translated into English by two independent bilingual experts to maintain accuracy and linguistic consistency.

In the familiarization phase, the research team repeatedly read through the transcripts to gain a deep understanding of participants' experiences. Initial coding was conducted inductively by two researchers working independently to allow for the emergence of concepts directly from the data, without the imposition of pre‐existing categories [18]. Following this, the researchers met to compare their codes [18]. Discrepancies were discussed openly and resolved through consensus. Where disagreement persisted, a third senior researcher was consulted to adjudicate and ensure intercoder reliability.

A preliminary codebook was developed after reviewing the first few transcripts and was iteratively refined throughout the analysis process [18]. As new codes emerged or existing ones were collapsed, the codebook evolved to reflect a more accurate representation of the data. Codes were then clustered into potential themes based on conceptual similarities. Thematic mapping techniques—such as visual matrices and code‐to‐theme charts—were used to organize relationships among codes, subthemes, and overarching themes [18].

Peer debriefing sessions were held at multiple stages of analysis [16]. These sessions provided an opportunity for team members not directly involved in coding to challenge interpretations, question assumptions, and help refine the emerging themes. These discussions enhanced the credibility and confirmability of the findings [16].

To further strengthen transparency and rigor, representative data excerpts and summary coding tables were used to trace the analytical path from raw data through codes to final themes [16]. Themes were reviewed and refined for internal consistency and external distinction, then clearly defined and interpreted in the context of existing literature on RMC.

Reflexivity was maintained throughout the process. Researchers engaged in reflexive journaling and regular team discussions to acknowledge and examine their own positionalities, professional roles, and potential biases [16]. These practices helped minimize subjective influence on data interpretation and supported a more grounded and authentic representation of participants' voices.

Data collection and analysis were conducted concurrently, allowing the team to monitor thematic saturation in real time [15]. Saturation was considered achieved when no new codes or themes emerged from the data [15]. This occurred by the 18th interview, but two additional interviews were conducted to confirm stability and completeness of the findings.

### 2.6 Ethical Consideration

Ethical approval was received from the Erbil Polytechnic University (Ref‐11336), and permission was granted from relevant authorities of the hospitals. Informed consent in verbal was obtained from the participants before conducting the interviews. They were assured of confidentiality, voluntary participation, and the right to withdraw at any stage without repercussions. Pseudonyms were used at the time of transcription, and identifiable information was omitted to ensure anonymity. No monetary or material compensation was offered to participants. This approach was intended to minimize potential coercion and ensure that participation was fully voluntary. All participants were informed that their involvement was entirely optional and would not affect the quality of care they received. As well as prior to participation, all eligible women were provided with a clear explanation of the study's purpose, procedures, and their rights, including the right to decline or withdraw at any time without consequences. Written informed consent was obtained from each participant before beginning the interview. For participants with limited literacy, the information was read aloud, and verbal consent was documented in the presence of a witness.

### 2.7 Trustworthiness/Rigor

Ensuring trustworthiness in qualitative research is essential for maintaining the rigor and credibility of findings. This study adheres to Ahmed (2024) criteria, focusing on credibility, dependability, confirmability, and transferability [16]. Credibility was enhanced through triangulation of data sources, member checking, and peer debriefing to validate findings. Dependability was ensured by maintaining an audit trail and employing independent coding by multiple researchers to enhance consistency. Confirmability was strengthened through reflexivity, bracketing, and transparent documentation of the research process. Lastly, transferability was supported by thick descriptions of the study setting and purposeful sampling to ensure findings are applicable in similar contexts [16].

## 3 Results

### 3.1 Demographics of Participants

The demographic profile of the participants (*n* = 20) reveals a predominantly young population, with ages ranging from 17 to 41 years and a mean age of 24.75 years. Educational attainment varied, with the largest proportion (40%) having completed secondary school, followed by primary school graduation (30%) and 15% being able to read and write, while 5% were illiterate. Only one participant (5%) had attained higher education, indicating relatively low educational levels overall. In terms of employment status, more than half (55%) of the participants were unemployed, while 30% were self‐employed and 5% reported contract employment. The number of children per participant ranged from 1 to 3, with the majority having two children (45%) or one child (40%). Residency data showed that 50% of participants lived in urban areas, 30% in suburban areas, and 20% in rural areas. Income levels were predominantly in the middle‐income category (65%), with 20% reporting low‐income and 10% indicating high‐income status. All participants relied on public sector healthcare services (Table 1).

**Table 1:** Demographic profile of participants.

No	Age	Educational level	Occupation	No. of children:	Residency	Income level	Healthcare experience
P1	17	Able to read and write	Un employed	1	Rural	Low	Public sector
P2	19	Primary school graduation	Un employed	1	Urban	Middle	Public sector
P3	24	Secondary school graduation	Un employed	1	Urban	Middle	Public sector
P4	27	Primary school graduation	Un employed	1	Suburban	Middle	Public sector
P5	32	Primary school graduation	Contract employment	2	Suburban	Middle	Public sector
P6	21	Secondary school graduation	Self‐employed	2	Urban	Middle	Public sector
P7	23	Able to read and write	Self‐employed	2	Urban	Middle	Public sector
P8	25	Secondary school graduation	Un employed	2	Urban	Middle	Public sector
P9	27	Primary school graduation	Un employed	2	Rural	Middle	Public sector
P10	41	Higher education	Self‐employed	3	Urban	High	Public sector
P11	22	Able to read and write	Self‐employed	3	Rural	Middle	Public sector
P12	36	Secondary school graduation	Un employed	1	Urban	High	Public sector
P 13	31	Secondary school graduation	Un employed	2	Urban	Middle	Public sector
P 14	27	Primary school graduation	Self‐employed	3	Suburban	Middle	Public sector
P 15	19	Able to read and write	Un employed	2	Suburban	Middle	Public sector
P 16	17	Illiterate	Un employed	1	Urban	Low	Public sector
P 17	18	Primary school graduation	Un employed	1	Rural	Low	Public sector
P 18	19	Primary school graduation	Un employed	1	Rural	Low	Public sector
P 19	27	Secondary school graduation	Un employed	1	Urban	Low	Public sector
P 20	29	Secondary school graduation	Un employed	2	Urban	Middle	Public sector

### 3.2 Thematic Analysis Results

Thematic analysis of the interviews revealed six major themes that encapsulate the perspectives of Kurdish postnatal women regarding RMC: respect and dignity, gentleness and supportive care, clear and respectful communication, privacy, family presence, and cultural and religious sensitivity.

#### 3.2.1 Respect and Dignity

Participants emphasized the need to be treated with respect and without discrimination, particularly in relation to socioeconomic status. Several women expressed distress over verbal abuse, judgment, and neglect, urging healthcare providers to adopt a more compassionate and respectful approach during childbirth.

- *“I didn't want to face discrimination because of my low economic status. I wanted to be treated with respect, without being shouted at.”* (Woman 6)
- *“I didn't have money, which is why I'm here. If I had money, I would go to the private sector where no one would shout or rush me during childbirth. I don't want to be neglected; please let me know when the baby is born and communicate with me gently.”* (Woman 11)
- *“Don't insult me or my companion, and don't allow too many vaginal examinations by different midwives.”* (Woman 13)
- *“Don't judge me or slap me. Please communicate with me and respect me and my family.”* (Woman 20)

#### 3.2.2 Gentleness and Supportive Care

Women strongly expressed the need for kindness, patience, and emotional support from healthcare providers. They preferred calm and understanding midwives, emphasizing the impact of verbal encouragement and a reassuring presence on their childbirth experience.

- *“I wanted respectful and caring treatment during childbirth. I preferred midwives who were kind, calm, and patient. Instead of being rushed or yelled at, I wanted gentle communication. This made me feel safer and more supported during birth.”* (Woman 1)
- *“I wanted to be listened to, have someone hold my hand, and be cared for with genuine compassion. I didn't want to be yelled at but rather treated with kindness and understanding.”* (Woman 2)
- *“Midwives, listen to me and show mercy. Don't keep me waiting for a long time without providing any care during labor.”* (Woman 14)
- *“Respectful care to me means a warm welcome. Don't neglect me or leave me alone in the delivery room while the midwife goes to the staff room.”* (Woman 16)

#### 3.2.3 Clear and Respectful Communication

A recurring concern among participants was the lack of clear and respectful communication from healthcare providers. Many women expressed frustration over being left uninformed about the progress of labor and facing verbal threats or abuse. They highlighted the importance of gentle, informative, and reassuring communication.

- *“Answer my questions honestly, keep me informed about the progress of labor, and don't use verbal abuse or shout at me.”* (Woman 15)
- *“I appreciate receiving verbal praise and encouragement from midwives during labor, especially when they have a warm and respectful manner.”* (Woman 12)
- *“The delivery room staff should communicate with me respectfully. I'm in pain, so help me—don't scream at me.”* (Woman 18)
- *“Don't threaten me with a cesarean section or tell me about a dead baby if I'm not pushing well. The delivery room staff should be kind to me.”* (Woman 10)

#### 3.2.4 Privacy and Consent

Privacy during labor and medical procedures, particularly vaginal examinations, was a major concern. Participants emphasized the importance of seeking consent before examinations, limiting their frequency, and ensuring privacy through physical barriers such as curtains.

- *“Please take permission before conducting a vaginal examination and do not do it frequently. Respect me by preserving my privacy.”* (Woman 8)
- *“I want to protect my privacy, but unfortunately, every staff member comes into the delivery room, and I feel ashamed. Please use a curtain—it would help.”* (Woman 17)

#### 3.2.5 Family Presence and Emotional Support

Many women valued the presence of family members during labor, particularly sisters or other trusted companions, as a source of emotional support and security. Some expressed that they felt more respected and comforted when a relative was present.

- *“Having my family (my sister) present during labor made me feel respected. I also emphasized that the health workers didn't shout at me.”* (Woman 3)
- *“Don't leave me alone. The nurses were usually with me in the same room, but I want my companion (my sister) to stay with me during labor and delivery. Please respect me by not shouting.”* (Woman 7)

#### 3.2.6 Cultural and Religious Sensitivity

Cultural and religious considerations played a significant role in how women perceived RMC. Some women expressed a strong preference for midwives to offer prayers for a smooth delivery, while also requesting gentle treatment and avoidance of harsh language.

- *“I expressed a strong preference for midwives to pray for me to have an easy birth and to avoid yelling at me during labor.”* (Woman 4)
- *“Pray for me. Be gentle with me—don't shout and don't slap me.”* (Woman 9)

The study highlights the critical importance of respect, kindness, and clear communication in maternity care. Women overwhelmingly sought compassionate and patient‐centered interactions, emphasizing their need for privacy, informed decision‐making, and the presence of a supportive companion. Additionally, socioeconomic disparities and cultural considerations played a crucial role in shaping their experiences of RMC. Addressing these concerns through midwife training, improved communication strategies, and policy changes can enhance maternal satisfaction and trust in healthcare services.

## 4 Discussion

This study explored Kurdish postnatal women's perceptions, preferences, and experiences regarding RMC, with particular attention to communication, emotional support, privacy, and cultural sensitivity. The findings highlight that women place great value on compassionate, respectful, and patient‐centered care during childbirth. Based on thematic analysis, we discuss our findings across five interrelated themes.

RMC is widely recognized as a fundamental human right, ensuring that women receive care that upholds their autonomy, dignity, emotional well‐being, and cultural preferences. This includes their right to have a companion, make informed decisions, and engage in cultural or religious practices during childbirth [1, 2].

A recurring theme was the critical need for respectful treatment regardless of socioeconomic status. Women reported verbal abuse, shouting, and being judged or neglected, particularly when they lacked financial means. These behaviors were not only distressing but also shaped women's expectations and desires for care that affirms their basic dignity. These findings are consistent with studies from South Asia, where mistreatment of marginalized women in public maternity wards has been well documented [19]. While our study did not directly assess women's satisfaction or trust in the healthcare system, the findings illustrate how experiences of disrespect influence expectations for future care and underscore the need for improved standards in provider conduct.

Disrespect and mistreatment during childbirth not only violate fundamental human rights but also compromise maternal health outcomes and psychological well‐being. Women who experience verbal abuse, neglect, or mistreatment during labor are less likely to seek facility‐based care in subsequent pregnancies, increasing the risk of maternal and neonatal complications. Upholding RMC principles is therefore critical in fostering a safe, supportive, and empowering childbirth experience [3, 20, 21].

Poor communication was widely reported. Women described feeling uninformed about labor progress and sometimes faced coercive threats (e.g., being warned about a cesarean or fetal death). Conversely, they appreciated providers who gave updates, explained procedures, and offered words of encouragement.

This echoes prior research in Iraq and similar contexts, which show that communication—both verbal and non‐verbal—is a key determinant of childbirth experience and satisfaction [22]. Cultural norms in the Kurdistan Region, where authority is rarely questioned, may intensify the impact of poor communication on women's sense of agency [23]. Contributing factors may include high workload, provider burnout, and a lack of training in patient‐centered communication [24, 25].

Another study in 2020 in Iraqi Kurdistan, where 41.6% of 1196 women surveyed reported dissatisfaction with delivery room communication [22]. The link between satisfaction and both verbal and nonverbal communication suggests that improving healthcare providers' communication skills could enhance delivery room care. Similar research consistently shows that clear, supportive interactions from healthcare staff positively impact women's childbirth experiences [22].

An interventional study conducted in the Kurdistan Region of Iraq demonstrated that implementing RMC and effective communication (EC) during labor and delivery can significantly reduce postpartum depression rates [26]. The study also highlighted how compassionate, patient‐centered care enhances overall childbirth satisfaction, reinforcing the need for structured RMC interventions in maternity wards [26].

Women consistently expressed a preference for gentle, calm, and kind midwives. Many noted how a soft tone, holding hands, or being emotionally present created a feeling of security during labor. The absence of this care was described as neglect [27].

Such preferences reflect the emotional vulnerability of women during childbirth and the importance of interpersonal behavior. In our context, the lack of emotional support may also reflect systemic constraints and an undervaluation of “soft skills” in clinical care.

Concerns about privacy were raised in relation to repeated vaginal examinations, lack of curtains, and frequent staff interruptions. Women felt that their bodily autonomy was compromised when consent was not sought or when procedures were conducted in view of others, which contributed to increased stress and anxiety during labor. These findings align with global research emphasizing the importance of environmental and procedural privacy in shaping childbirth experiences, as the lack of basic structural provisions such as curtains has been shown to undermine dignity and elevate stress during labor [28, 29, 30, 31]. From a broader perspective, academic literature highlights that the physical and social environment plays a fundamental role in the childbirth process, as unfamiliar surroundings combined with a lack of privacy can increase fear, anxiety, and pain perception [32]. Privacy in childbirth has been defined as a woman's ability to refuse examinations, control who enters the room, move freely, choose labor positions, and experience uninterrupted bonding with her newborn, underscoring its central role within RMC frameworks [33, 34].

Many participants emphasized the value of having a trusted companion—typically a sister—present during labor, which greatly enhanced their emotional comfort and sense of dignity. Conversely, some women described feeling isolated, anxious, or even traumatized when left alone during childbirth. This finding aligns with a growing body of research highlighting the positive impact of continuous support during labor. A Cochrane review by Bohren et al. (2017) found that women who received continuous labor support were more likely to have spontaneous vaginal births, less likely to require intrapartum analgesia, and reported more positive childbirth experiences [35]. In culturally collectivist societies like the Kurdistan Region, family presence may carry even deeper emotional significance. For instance, Kabakian‐Khasholian and Portela (2017) emphasized that in Middle Eastern contexts, where familial bonds are particularly strong, emotional support from trusted relatives can reduce anxiety and foster feelings of safety [36]. Similarly, a qualitative study from Jordan reported that women associated the presence of a close female relative with greater respect from healthcare staff and a more empowered birthing experience [37]. In Iran, family support has been shown to significantly affect maternal satisfaction and perceived respect during childbirth [38]. These comparisons underscore that facilitating family presence—especially of female relatives—may be a culturally attuned, low‐cost strategy to enhance RMC in the region.

While our findings are aligned with existing literature on disrespect and abuse during childbirth, they offer important insights into the Kurdish context. In a society marked by strong family bonds, traditional gender roles, and limited health system resources, the delivery of respectful care requires not only technical training but also cultural humility and structural investment.

The study findings support the need for targeted interventions, such as mandatory RMC training, staff wellness programs to address burnout, and institutional reforms to ensure privacy, informed consent, and continuous labor support. These reforms are essential for improving maternal experiences and ensuring that care is delivered in accordance with global standards and local expectations.

Despite the valuable insights provided by this study, several limitations must be acknowledged. First, the study relied on a qualitative design, which, while effective in capturing in‐depth experiences, limits generalizability to broader populations. The findings reflect the perspectives of Kurdish postnatal women in a specific public hospital setting, and experiences may differ in private healthcare facilities or rural areas. Second, the study relied on self‐reported data, which may be influenced by recall bias or social desirability bias, as participants may have underreported or overemphasized certain aspects of their maternity care experiences. Third, the timing of data collection (within 1–3 h post‐delivery) may have influenced responses, as women might have been emotionally overwhelmed or physically exhausted, potentially affecting their ability to articulate their experiences fully. Fourth, while efforts were made to ensure a diverse sample, variations in literacy levels, socioeconomic backgrounds, and prior childbirth experiences may have influenced participants' perceptions of RMC. Another limitation is that interviewing women within hours after delivery may introduce emotional or social desirability bias due to the heightened emotional state immediately postpartum. However, we attempted to mitigate this by ensuring that interviews were conducted in a calm, private setting, with trained interviewers using a supportive and non‐judgmental approach. Participants were reminded that their responses would remain confidential and would not affect their care. While emotional salience may influence perceptions, it may also enhance the richness and immediacy of reflections on maternity care.

Finally, the study focused exclusively on women's perspectives, and future research should incorporate the views of healthcare providers, policymakers, and birth companions to gain a comprehensive understanding of systemic challenges in maternity care. Addressing these limitations in future studies will enhance the depth and applicability of research on RMC.

## 5 Conclusion

This study highlights the urgent need for improving maternity care policies to align with the respect and dignity, cultural and religious sensitivity, psychological and emotional needs, and physical autonomy of Kurdish postnatal women. The lived experiences shared by participants expose ongoing challenges related to mistreatment, verbal abuse, lack of privacy, and inadequate communication during childbirth issues that undermine both maternal trust and the perceived quality of care. To improve maternity care in the Kurdistan Region, institutional commitment is needed to translate RMC principles into practice. This includes structured training in effective communication, regulation of provider behavior, policies that allow family support during labor, and reinforcement of privacy safeguards such as informed consent and reduced unnecessary examinations. Future studies should expand the evidence base by including perspectives from health workers, exploring provider constraints, and evaluating the effectiveness of RMC interventions. Addressing these areas will not only improve maternal experiences but also advance broader goals of health equity, human rights, and trust in the healthcare system.

## Author Contributions

**Khalat Karwan Fares** and **Hamdia Mirkhan Ahmed:** writing – review and editing, writing – original draft, visualization, validation, software, resources, project administration, methodology, investigation, formal analysis, data curation, conceptualization. **Kochr Ali Mahmood** and **Hiwa Abdulrahman Ahmad:** writing – review and editing, writing – original draft, validation, investigation, formal analysis, data curation, conceptualization. **Sirwan Khalid Ahmed** and **Moustaq Karim Khan Rony:** writing – review and editing, writing – original draft, visualization, validation, supervision, software, project administration, methodology, formal analysis, data curation, conceptualization. All authors have read and approved the final version of the manuscript.

## Funding

The authors received no specific funding for this work.

## Disclosure

The lead author Sirwan Khalid Ahmed, Moustaq Karim Khan Rony affirms that this manuscript is an honest, accurate, and transparent account of the study being reported; that no important aspects of the study have been omitted; and that any discrepancies from the study as planned (and, if relevant, registered) have been explained.

## Ethics Statement

Ethical approval for this study was obtained from the Institutional Review Board of the Erbil Polytechnic University (Ref‐11336).

## Conflicts of Interest

The authors declare no conflicts of interest.

## Data Availability

The data sets used and/or analyzed during the current study are available from the corresponding author upon reasonable request.
